# Supplementary material for: jsPCA: fast, scalable, and interpretable identification of spatial domains and variable genes across multi-slice and multi-sample spatial transcriptomics data
Source: Bioinform Adv. 2026 Jul 25;6(1):vbag206. doi: 10.1093/bioadv/vbag206 (PMC13532759; doi:10.1093/bioadv/vbag206)
Supplement: Supplementary file 1 [file vbag206_supplementary_data.pdf]

Supplementary Material

jsPCA: fast, scalable, and interpretable identification of spatial domains and variable genes across multi-slice and multi-sample spatial transcriptomics data

Ines Assali[1,*], Paul Escande[2], Franck Picard[3], Paul Villoutreix[1,*]

[1] Aix-Marseille Université, MMG, Inserm U1251, Turing Centre for Living Systems, Marseille, France

[2] Institut de Mathématiques de Toulouse, Université de Toulouse, CNRS, Toulouse, France

[3] Université de Lyon, ENS de Lyon, Université Claude Bernard, CNRS, INSERM, Lyon, France

[*] Corresponding authors: ines.assali@univ-amu.fr, paul.villoutreix@univ-amu.fr

---

1 Supplementary Text 1 - Computation of jsPCA for large matrices

To compute jsPCA eigenvectors for a collection of ST datasets, i.e. a collection of expression matrices and normalized adjacency matrices $\{X_j, L_j\}$, we need to perform a joint diagonalization of the corresponding spatial covariance matrices. This can be summarized by finding the common set of eigenvectors P such that

$$A_j := \frac{1}{2n_j} X_j^T (L_j + L_j^T) X_j \simeq P D_j P^T,$$

The matrices P and $\{D_j\}$ can be obtained by solving

$$\begin{aligned} \min_{P, D_j} \quad & \frac{1}{2} \sum_j \| P D_j P^T - A_j \|^2 \\ \text{subject to} \quad & P \in \mathbb{R}^{p \times p}, \quad P^T P = P P^T = \mathrm{I}_p \\ & \lambda_j \in \mathbb{R}^p, \quad D_j = \mathrm{diag}(\lambda_j) \end{aligned}$$

Given the large size of the matrices handled in ST, the joint diagonalization of the spatial covariances matrices $\{A_j\}$ cannot be obtained by classical approaches such as Jacobi-like techniques because they require too much memory [Cardoso and Souloumiac, 1996]. Instead, we implemented the following approach.

1.1 Independent truncated diagonalizations

First, the truncated diagonalizations of order K of each A_j are computed as $A_j \simeq P_j S_j P_j^T$. In our experiments, we use values of K ranging from 10 to 50, hence $K \ll p$. For $K \ll p$ the truncated diagonalization can be efficiently computed using an Implicitly Restarted Lanczos Method which is implemented in the scipy library [Lehoucq et al., 1998]. The key feature of this method is its *matrix-free* operation meaning that it only uses matrix-vector products with A_j and does not require a matrix built in memory. However, since p is large and the matrices A_j are dense, directly computing the eigensystem from A_j is particularly inefficient (considering the building time and the dense matrix-vector products). Fortunately the matrices X_j and L_j are very sparse. Implementing an efficient matrix-vector product with A_j can therefore be performed by successive sparse matrix-vector products, that is implementing $A_j u = \frac{1}{2n_j} X_j((L_j + L_j^T)(X_j^T u))$ for $u \in \mathbb{R}^p$.

1.2 Joint approximate diagonalization

Given the eigensystems $\{(P_j, D_j)\}_j$, the joint diagonalization can now be modeled as

$$\begin{aligned} \min_{P, D_j} \quad & \frac{1}{2} \sum_j \|P D_j P^T - P_j S_j P_j^T\|^2 \\ \text{subject to} \quad & P \in \mathbb{R}^{p \times K}, \quad P^T P = \mathrm{I}_K \\ & \lambda_j \in \mathbb{R}^K, \quad D_j = \mathrm{diag}(\lambda_j) \in \mathbb{R}^{K \times K} \end{aligned}$$

With an argument similar to [Congedo et al., 2010] this problem can be shown to be equivalent to

$$\begin{aligned} \min_{P} \quad & -\frac{1}{2} \sum_j \left\|\mathrm{diag}\left(P^T P_j D_j P_j^T P\right)\right\|^2 \\ \text{subject to} \quad & P \in \mathbb{R}^{p \times K}, \quad P^T P = \mathrm{I}_K \end{aligned}$$

which can be solved by a gradient descent on the Stiefel manifold [Theis et al., 2009, Townsend et al., 2016] (modeling the orthogonality constraint $P^T P = \mathrm{I}_K$). Following again [Congedo et al., 2010], an initialization close to a minimizer can be obtained by computing the K largest left singular vectors of the matrix $(P_1 S_1, \ldots, P_j S_j, \ldots)$ (i.e. concatenating the columns of the $\{P_j S_j\}$) and by letting $D_j = \mathrm{diag}(P^T P_j S_j P_j^T P)$. In our experiments, we performed 100 steps for the gradient descent.

To further justify this choice, we performed an additional convergence analysis of the joint diagonalization step (Figure S9). Specifically, we tracked the evolution of the cost $J^{(t)}$, which is equal to the quantity $-\frac{1}{2} \sum_j \left\|\mathrm{diag}\left(P^T P_j D_j P_j^T P\right)\right\|^2$ evaluated at iteration t of the gradient descent and the normalized convergence gap $(J^{(t)} - J^\star)/(J^{(0)} - J^\star)$ across iterations, for both random and SVD initializations. The experiment was performed on two slices from the DLPFC dataset (151673 and 151674), comprising 3639 and 3673 spots respectively, with 15026 genes per slice. The results show that the cost decreases rapidly during the first few iterations and then reaches a stable plateau. In particular, most of this decrease occurs during the first 100

iterations, after which the improvement becomes negligible. While both initialization methods reach similar final values, the initial point provided by the SVD is very close to the optimum, roughly saving 4000 iterations compared to a random initialization. This is substantiated by the normalized convergence curve showing that the steepest descent only marginally improves the cost of the current iterate. For this reason, we use SVD initialization in all experiments together with 100 gradient descent iterations.

2 Supplementary Text 2 - Formal comparison between jsPCA and spatial-PCA like approaches

We review in this section the differences between various formulations of *spatial PCA* applied to spatial transcriptomics that have been proposed recently, with harmonized notations.

We consider a matrix of gene expression measurements for p genes in n spatial locations of a tissue, denoted by $X \in \mathbb{R}^{n \times p}$. These measurements have spatial coordinates that are denoted by $S = \{s_i \in \mathbb{R}^k, i \in \{1, .., n\}\}$ where $k = 2$ or 3.

Recall the variational formulation of PCA, which consists in finding a d-dimensional projection $Z \in \mathbb{R}^{n \times d}$ such that:

$$\min_{Z,W} ||X - ZW^T||_2^2 \text{ , with } W^T W = I \tag{1}$$

This optimization problem can be solved analytically. The analytical solution leads to $Z = XW$ where W represents the top-d eigenvectors of the empirical covariance matrix $X^T X$.

PCA however does not take into account the spatial information S. Several methods have been proposed to incorporate spatial information in the context of spatial transcriptomics data analysis. We will distinguish between two families of approaches, the approaches based on the distance between spots and the approaches based on a spatial graph. In that case, the locations are turned into an undirected graph with the nearest neighbor algorithm, we denote by C the corresponding adjacency matrix. $C_{i,j} = 1$ if j is in the neighborhood of i, 0 otherwise.

2.1 Distance-based spatial PCA

2.1.1 Spatially aware dimension reduction for spatial transcriptomics [Shang and Zhou, 2022]

The expression matrix X is assumed to have been normalized through variance stabilizing transformation and further scaled for each gene to have zero mean and unit standard deviation.

The goal is to infer a $n \times d$ factor matrix Z that represents a low dimensional embedding of X. The approach is based on the following latent factor model:

$$X = C_v B + Z W^T + E \tag{2}$$

where C_v is an $n \times q$ matrix of covariates, and B is a $q \times p$ matrix of corresponding coefficients, here q is assumed to be equal to 1. W is a $p \times d$ factor loading matrix and E is an $n \times p$ matrix of residual errors, with elements $E_{i,j}$ following independent normal distribution with mean zero and variance σ_0^2, $E_{i,j} \sim \mathcal{N}(0, \sigma_0^2)$.

W is assumed to have orthonormal columns such that $W^T W = I$. For the d factors Z_l, i.e. the vectors of dimension n composing the factor Z matrix, the assumption is that they follow a multivariate normal distribution:

$$Z_l \sim \mathcal{N}(0, \Sigma_l) \tag{3}$$

with Σ_l, the $n \times n$ covariance matrix modeling the correlation among the spatial locations. Σ_l has for elements $\Sigma_l(i, i') = \sigma_0^2 \tau_l K(s_i, s_{i'})$ with Gaussian kernel $K(s_i, s_{i'}) = \exp\left(-\frac{\|s_i - s_{i'}\|^2}{\gamma}\right)$. τ_l and γ are two hyperparameters controlling the scale of the factor value relative to the residual error and the strength of the spatial correlation, respectively. γ is set numerically, in relation to the range of values in the data. $\tau_l = \tau$ is assumed constant across factors.

Maximum likelihood-based inference predicts the factor loading matrix W and the factor matrix Z together with the hyper-parameters (τ, σ_0^2).

The spatial domains are obtained by computing a clustering algorithm on Z, in this case Walktrap algorithm and Louvain algorithm for clustering analysis.

2.1.2 Randomized Spatial PCA (RASP): a computationally efficient method for dimensionality reduction of high-resolution spatial transcriptomics data [Gingerich et al., 2025]

This approach first computes a randomized PCA on the expression matrix X with rank d and computes W, the matrix containing the top-d principal components of the covariance. The data is projected on the principal components and stored in a matrix $Z = XW$. In a second step, the spatial information is taken into account with the connectivity matrix C of the nearest neighbors graph. From this connectivity matrix, a distance matrix D is computed with diagonal elements set to a value α. The matrix is inverted with a power β, and stored in a matrix $D_{\text{inv}} = (D^{-1})^\beta$ which is subsequently normalized column-wise. The final representation is obtained by computing the product $Z_s = D_{\text{inv}} Z = (D^{-1})^\beta X W$, which gives a spatially smoothed version of PCA-derived projection.

2.2 Graph-based spatial PCA

2.2.1 GraphPCA: a fast and interpretable dimension reduction algorithm for spatial transcriptomics data [Yang et al., 2024]

The expression matrix X is assumed to have been normalized by analytic Pearson residuals proposed by [Lause et al., 2021] and further scaled for each gene to have zero mean and unit standard deviation. Then, the top 3000 spatial variable genes of the SPARK package are selected.

GraphPCA takes physical distance into account by including a graph regularization to the variational PCA (1) leading to

$$\min_{Z,W} ||X - ZW^T||_2^2 + \frac{1}{2}\lambda \sum_{i,j \in C} C_{i,j} ||Z_i - Z_j||_2^2 \text{ , with } W^T W = I \tag{4}$$

where λ is a hyperparameter controlling the reconstruction error versus the smoothness of the projections on the underlying spatial neighborhood graph. This optimization problem can be rewritten as

$$\min_{Z,W} ||X - ZW^T||_2^2 + \lambda \text{Tr}(Z^T \text{Lap} Z) \text{ , with } W^T W = I \tag{5}$$

where $\text{Lap} = D - C$ is the Laplacian matrix of the spatial neighborhood graph, with D the diagonal matrix containing the degree of the nodes in the graph C.

The solution of equation (5) obtained with Lagrange multiplier is $Z^* = (I + \lambda \text{Lap})^{-1} XW$ and from this it follows that W^* is the collection of top-d eigenvectors of the graph-revised covariance matrix $X^T(I + \lambda \text{Lap})^{-1} X = X^T(I + \lambda(D - C))^{-1} X$.

2.2.2 PCA-based spatial domain identification with state-of-the-art performance [Schaub et al., 2025]

The preprocessing step for the expression data consists in normalization and log-transform per cell. In the same way as in jsPCA and graphPCA, for nichePCA [Schaub et al., 2025], the spatial information is encoded in a k-nearest neighbor algorithm. The joint spatial and expression representation is obtained by computing the mean gene expression of a cell and its neighbors. Classical PCA is computed on this joint representation to obtained a spatially aware low-dimensional representation.

2.3 Comparison with jsPCA

We have reviewed here recent approaches aimed at adapting classical PCA to spatially aware dimensionality reduction for spatial transcriptomics data. While they revolve around similar ideas, the mathematical formulations differ, leading to different numerical solutions.

jsPCA is based, for a single ST sample, on the diagonalization of the following spatial covariance which is obtained as the product of the spatial autocorrelation and the gene expression covariance:

$$A = \frac{1}{2n}X^T(L + L^T)X = \frac{1}{2n}X^T(D^{-1}C + (D^{-1}C)^T)X \tag{6}$$

The corresponding embedding is obtained as $Z = XW$, where W correspond to the top-d eigenvectors of the spatial covariance A.

From this analytical solution, we can directly see that this formulation is quite different from the one called spatialPCA in [Shang and Zhou, 2022], equations (2) and (3). Compared to jsPCA, spatialPCA is distance-based and relies on a Gaussian kernel for the spatial covariance. Moreover, the factors of spatialPCA [Shang and Zhou, 2022] can be found with maximum-likelihood based inference. Their complete derivation is quite heavy and can be found in [Shang and Zhou, 2022] supplementary material. It is noticeable that the influence of the spatial information is controlled by the hyperparameter τ.

Similarly for the method RASP [Gingerich et al., 2025], the optimal representation is obtained by $Z_s = (D^{-1})^{\beta}XW$, where D is a spatial distance matrix, and W corresponds to the eigenvectors of the PCA. Analytically, the solution is quite different from jsPCA and rely on a parameter β to control the influence of space, as well as several heuristic steps.

GraphPCA is closer to jsPCA as it is a graph-based approach, relying on a Laplacian regularization of the variational formulation of PCA [Belkin and Niyogi, 2003]. The corresponding graph-revised covariance matrix reads $X^T(I + \lambda(D - C))^{-1}X$, to be compared with the spacial covariance of jsPCA in equation (6). With this rewriting of the two covariance matrices, it is clear that the two approaches have rather different grounds.

Finally, NichePCA computes the mean of the expression of spatial location and its direct neighbor as the new feature on which to apply PCA. Although it exploits the direct neighbors information, this approach does not capture the full extent of cross terms that jsPCA captures by computing the spatial autocorrelation on the nearest neighbors graph.

3 Supplementary Text 3 - Statistical significance analysis of jsPCA performance

To assess whether the observed differences could be explained by chance, we performed statistical comparisons between jsPCA and the other methods across DLPFC and MOSTA datasets using both parametric (paired t-test) and non-parametric (Wilcoxon signed-rank) tests, based on ARI and NMI scores. The detailed results are provided in Tables S2-S7 and Figures S1-S3.

The results show that jsPCA consistently outperforms several methods, including Leiden, Louvain, NichePCA, and Spaceflow, with statistically significant differences and large effect sizes for both ARI and NMI. These improvements are stable across datasets and are confirmed by both statistical tests. When compared to stronger baselines such as BASS, STAGATE, GraphPCA, and SpatialPCA, jsPCA achieves overall competitive performance, and in several cases outperforms

them. In particular, jsPCA shows significant gains on the MOSTA dataset, while the differences are smaller and sometimes not statistically significant on the DLPFC dataset. This indicates that jsPCA is able to match or exceed their performance depending on the dataset.

In summary, these results indicate that the improvements observed with jsPCA are not due to random variation, but reflect a robust and competitive performance across different datasets and evaluation metrics.

3.1 Biological interpretation of jsPCA principal components

To interpret the signals captured by the principal components from a biological perspective, we examined how known marker genes, as reported in the DLPFC study [Maynard et al., 2021] are represented within each component (Figure S4 and Table S8). The spatial expression patterns of these marker genes in the ground truth data are shown in Figures S5-S6 and are used as an external reference for biological interpretation. The components capture distinct and meaningful spatial patterns. jsPC1 clearly reflects white matter (WM), as indicated by the strong contribution of MBP (rank 1) and MOBP (rank 6), while layer specific markers such as CUX2, RORB, and PCP4 are not enriched. jsPC2 highlights superficial cortical regions, which is supported by the enrichment of HPCAL1 (rank 2) and CUX2 (rank 38), consistent with layer 2 expression. In addition, SNAP25 (rank 9), a marker of gray matter (GM), further supports the cortical origin of this component. jsPC4 captures a mixed laminar signal, with contributions from both superficial and deep layers, as shown by HPCAL1 (rank 35, L2) and KRT17 (rank 24, L6). jsPC5 is primarily associated with layer 1 via AQP4 (rank 11), with a smaller additional contribution from deeper layers, as suggested by KRT17 (rank 27, L6). jsPC6 shows a weak but constant signal from layer 5, corroborated by PCP4 (rank 10) and a slight spatial enrichment in L5. In contrast, jsPC7 exhibits a strong, well-defined laminar structure dominated by the deeper layers (L4–L6), with high-ranking markers such as PCP4 (rank 1, L5), KRT17 (rank 5, L6), TRABD2A (rank 7, L1), and RORB (rank 12, L4), while CUX2 (rank 12411, L2) is not represented. The higher-order components, jsPC8 and jsPC9, are dominated by IGKC (rank 1 in jsPC8 and rank 4 in jsPC9) and reflect sparse, localized non-neuronal or immune-related signals. Finally, jsPC10 detects a weak mixed signal, with contributions from AQP4 (rank 26) and HBB (rank 27), suggesting a combination of non-neuronal expression or vascular origin, with a small contribution from layer 1. Overall, these results show that jsPCA decomposes the data into components that reflect both major anatomical structures, such as white matter and cortical layers, and secondary or non-neuronal sources of variation.

3.2 Effect of Spatial Refinement and Threshold Sensitivity

We evaluated clustering performance both before and after applying the spatial refinement step, as well as across different refinement thresholds. As shown in Figure S8, the jsPCA embedding already gives strong NMI and ARI scores without any refinement, indicating that the main structure is well captured on its own. Applying refinement leads to a small but consistent improvement, mainly by smoothing local inconsistencies, but the overall gains remain limited.

This suggests that most of the performance comes from the jsPCA embedding rather than this post-processing step. We also find that performance is robust to the choice of threshold. Moderate values (0.3–0.5) tend to give slightly higher median scores, while more stringent thresholds (≥ 0.7) do not provide further improvements. Overall, the variation across thresholds is small, indicating that the method does not depend on careful tuning of this parameter.

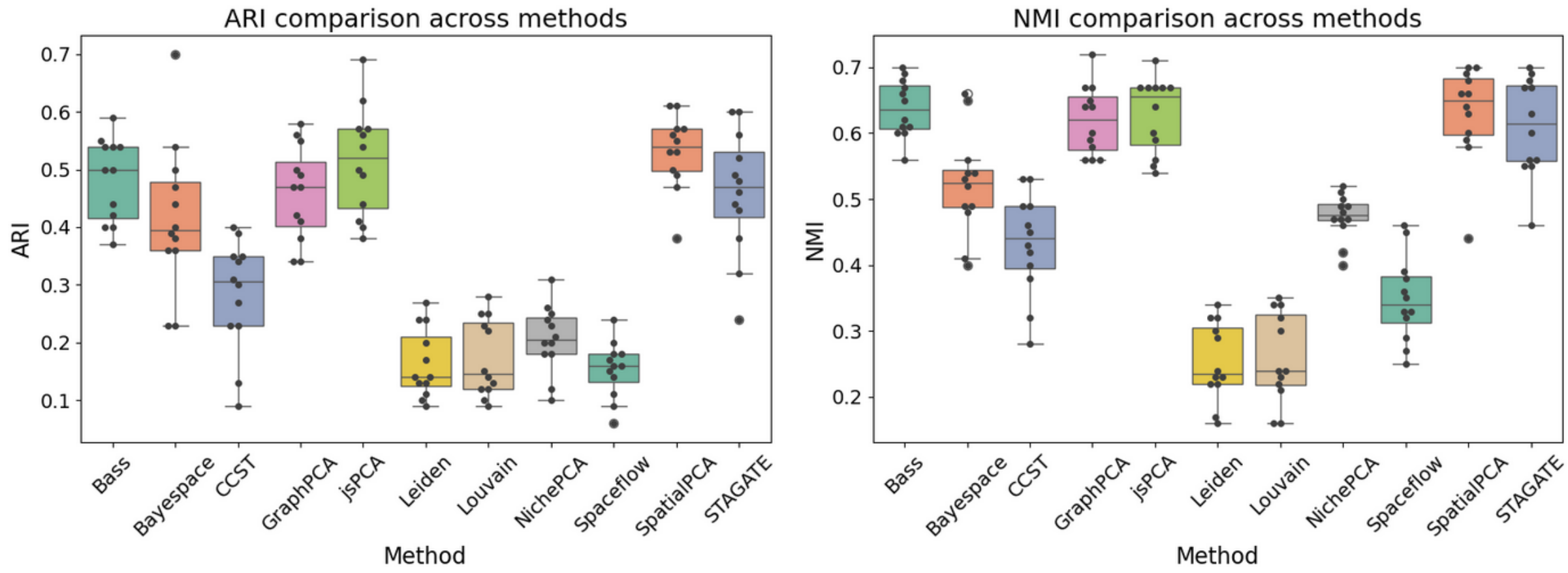

Figure S1: *ARI and NMI distributions across methods on the DLPFC dataset. Statistical differences between jsPCA and other methods were assessed using both a paired t-test and a Wilcoxon signed-rank test (see see Tables S2 and S3). Both tests lead to identical boxplot visualizations, so, a single figure is shown.*

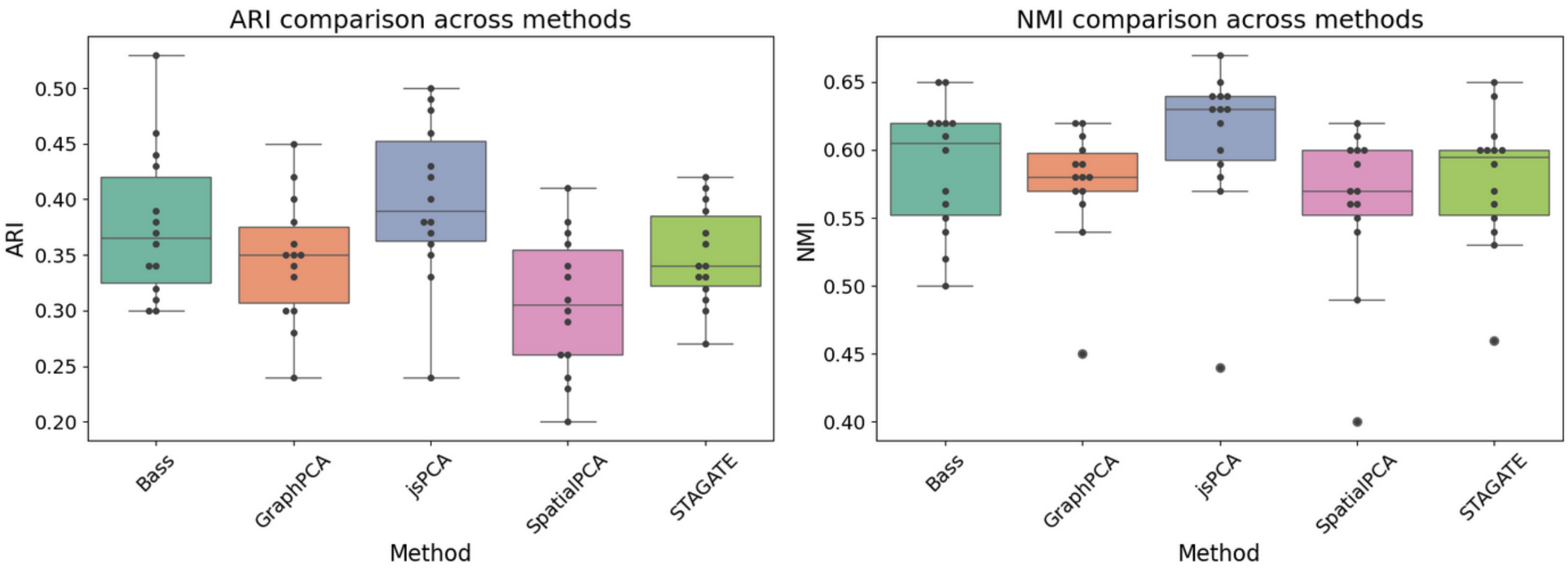

Figure S2: *ARI and NMI distributions across benchmark methods on the first 14 sagittal sections of the MOSTA dataset. Statistical differences between jsPCA and other methods were assessed using both a paired t-test and a Wilcoxon signed-rank test (see Tables S4 and S5). Both tests produce identical boxplot visualizations, so, a single figure is shown.*

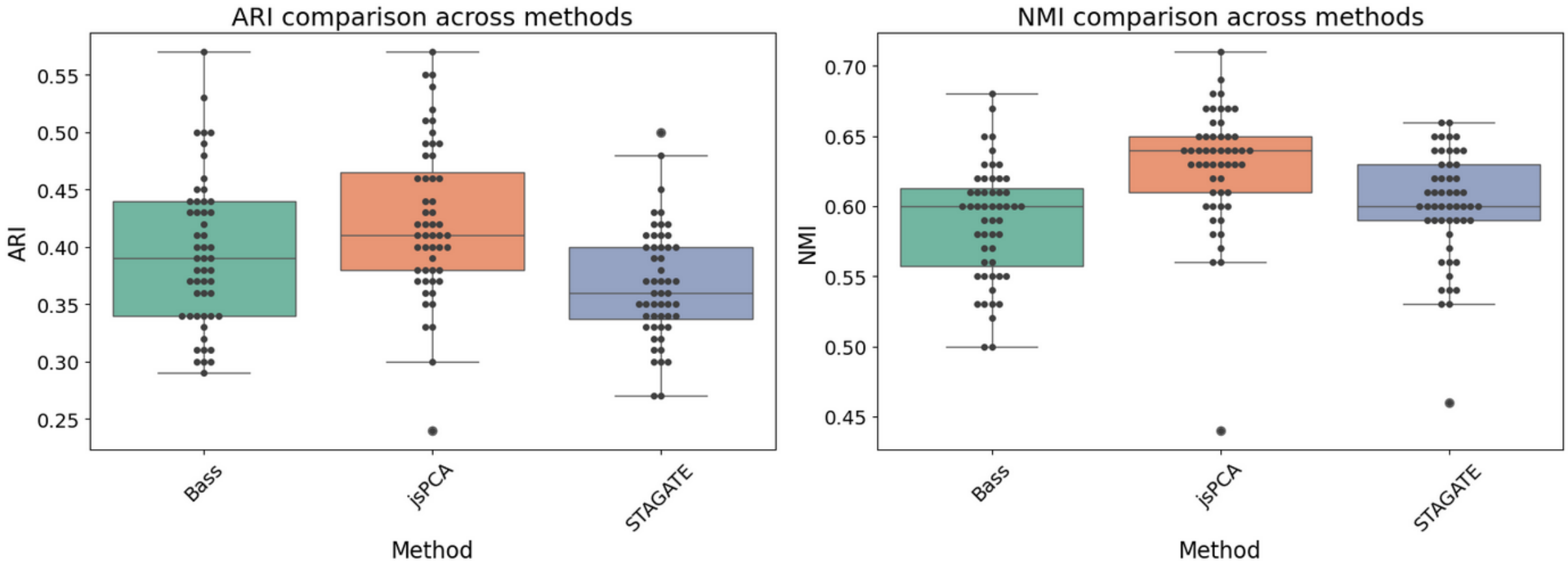

Figure S3: *ARI and NMI distributions across jsPCA, BASS, and STAGATE on the whole MOSTA dataset. Statistical differences between jsPCA and the other methods were assessed using both a paired t-test and a Wilcoxon signed-rank test (see Tables S6 and S7). Both tests produce identical boxplot visualizations, so, a single figure is shown.*

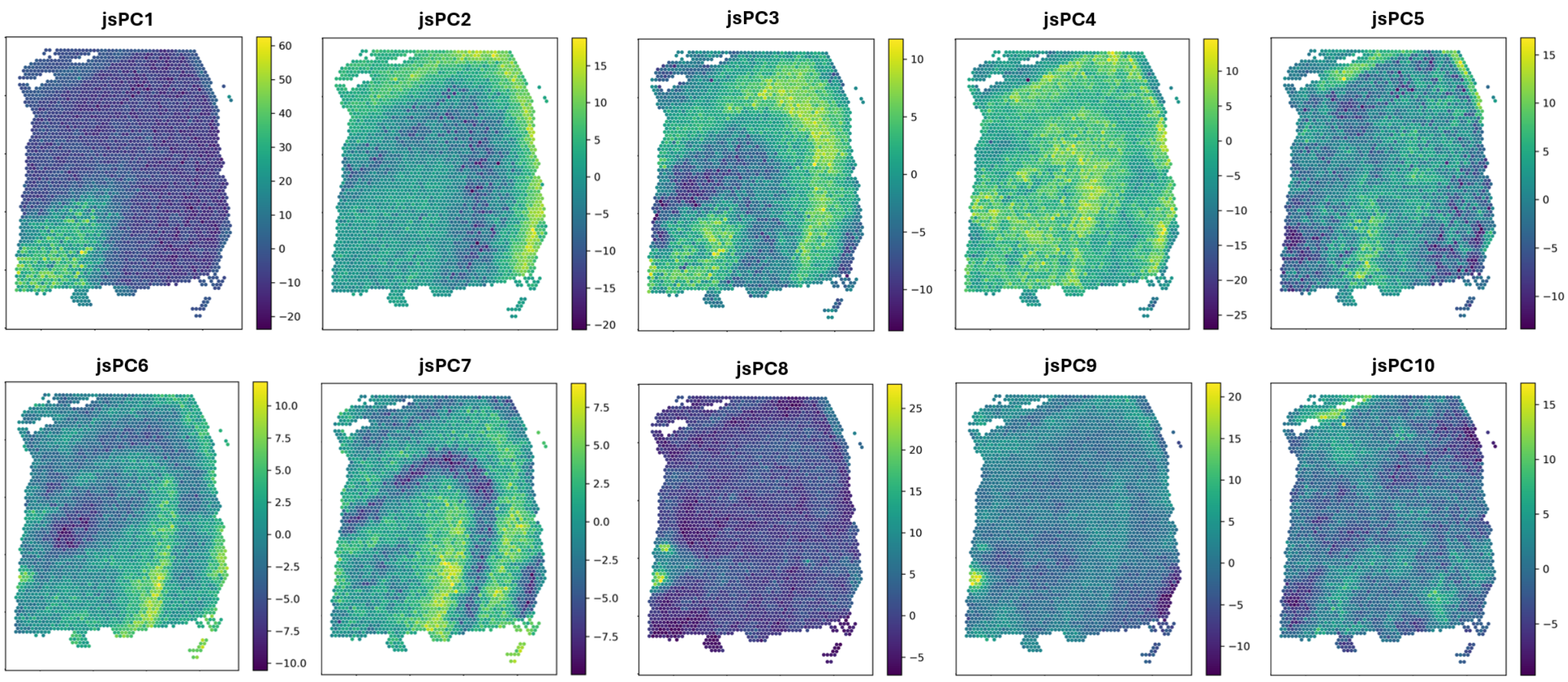

Figure S4: *Visualization of each of the 10 principal components of jsPCA for the slice 151673 of the DLPFC dataset.*

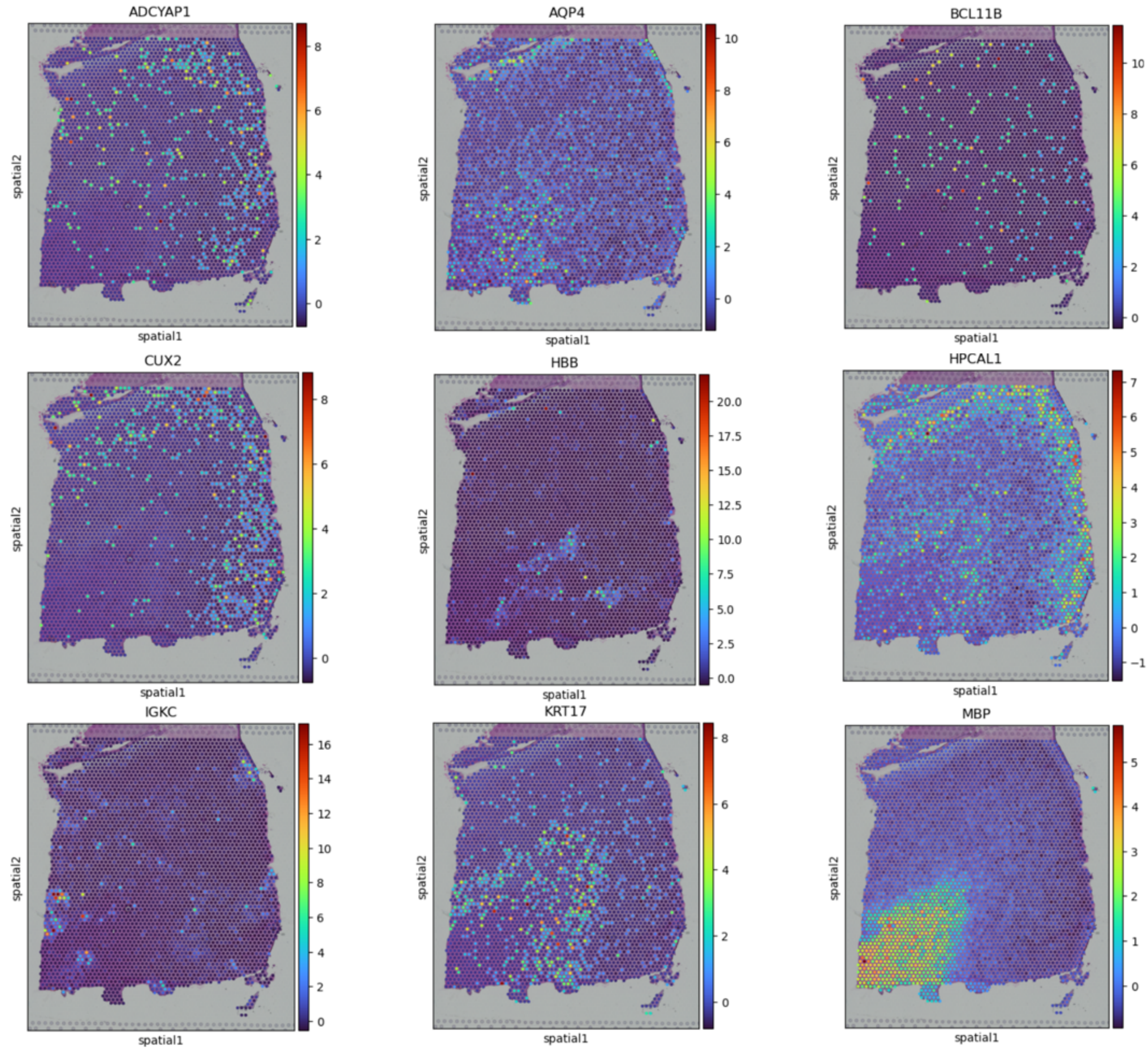

Figure S5: *Spatial expression patterns of marker genes (ADCYAP1, AQP4, BCL11B, CUX2, HBB, HPCAL1, IGKC, KRT17, MBP) in the ground truth tissue for slice 151673 of the DLPFC dataset.*

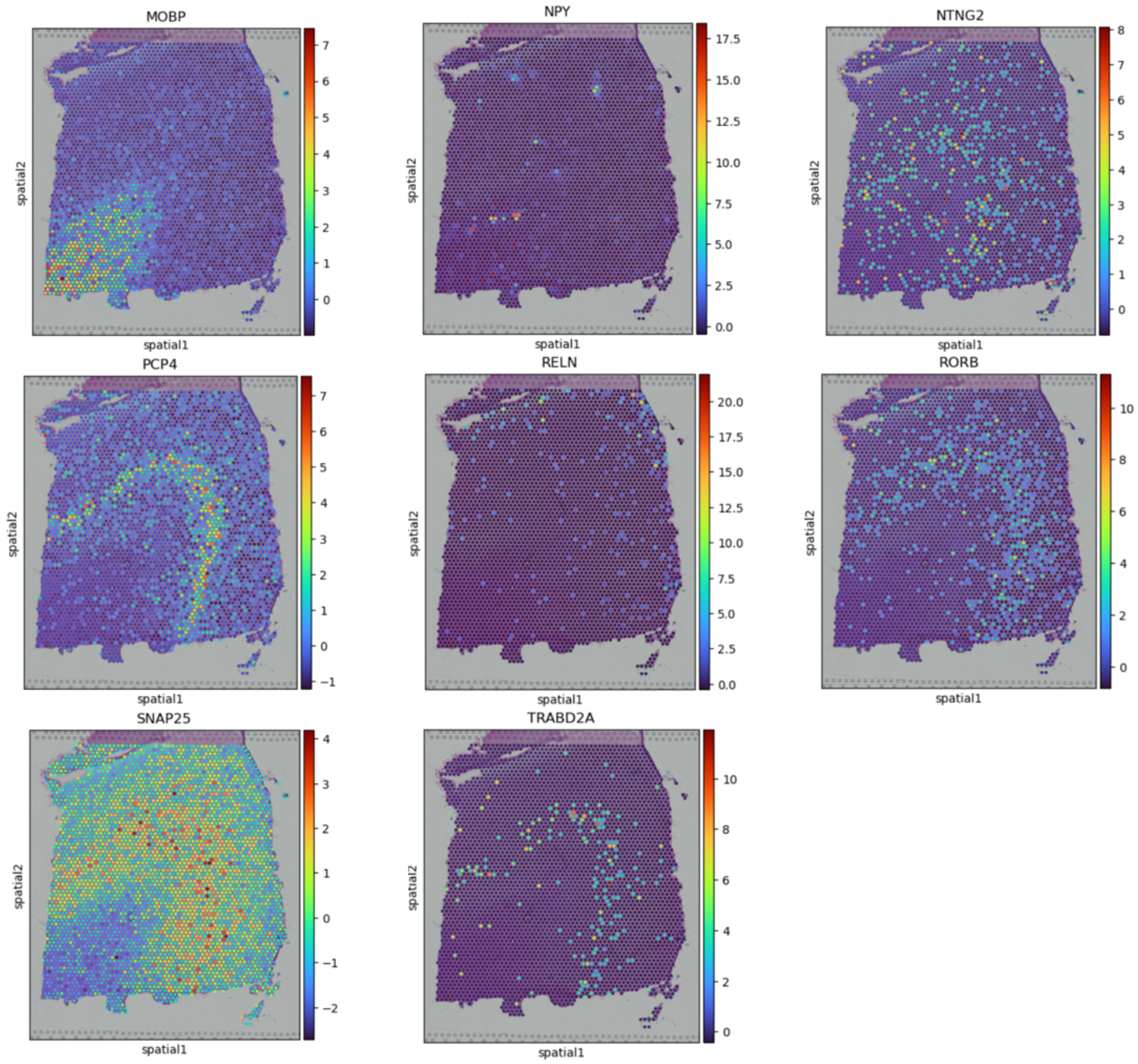

Figure S6: *Spatial expression patterns of marker genes (MOBP, NPY, NTNG2, PCP4, RELN, RORB, SNAP25, TRABD2A) in the ground truth tissue for slice 151673 of the DLPFC dataset.*

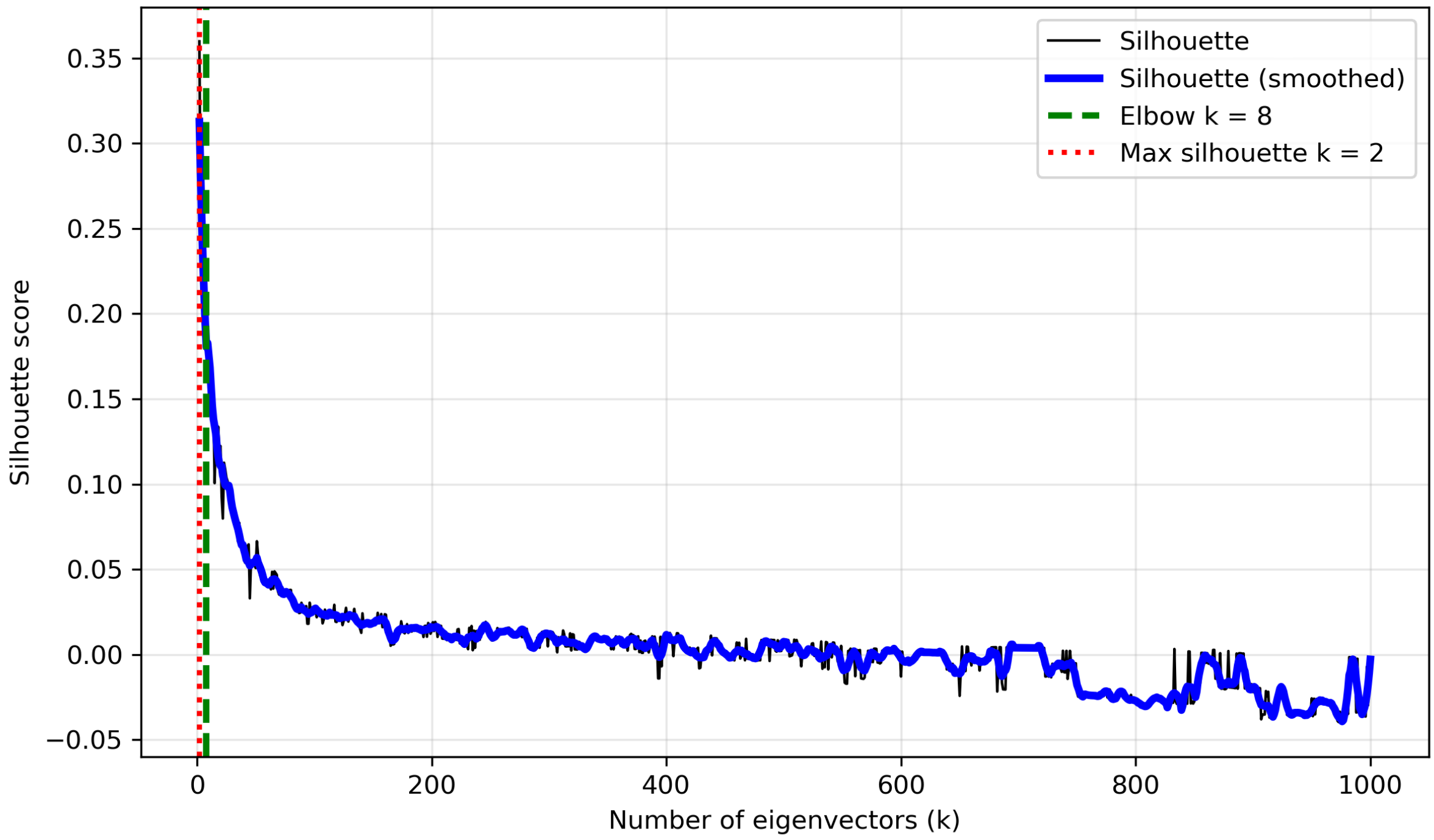

Figure S7: *Visualization of the silhouette score as a function of the number of retained eigenvectors (k) for the slice 151673 of the DLPFC dataset. The raw (black) and smoothed (blue) curves are shown, along with the selected elbow point (k=8, green dashed line) and the maximum silhouette value (k=2, red dotted line).*

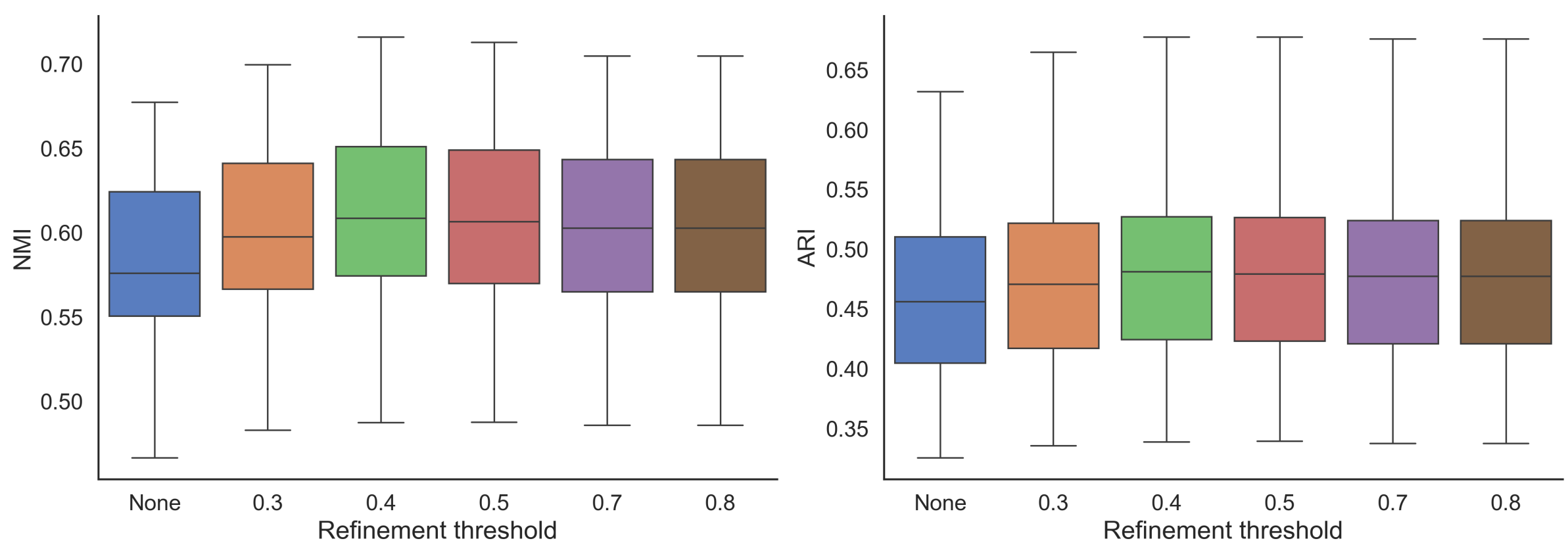

Figure S8: *Clustering performance across refinement thresholds for the DLPFC dataset.*

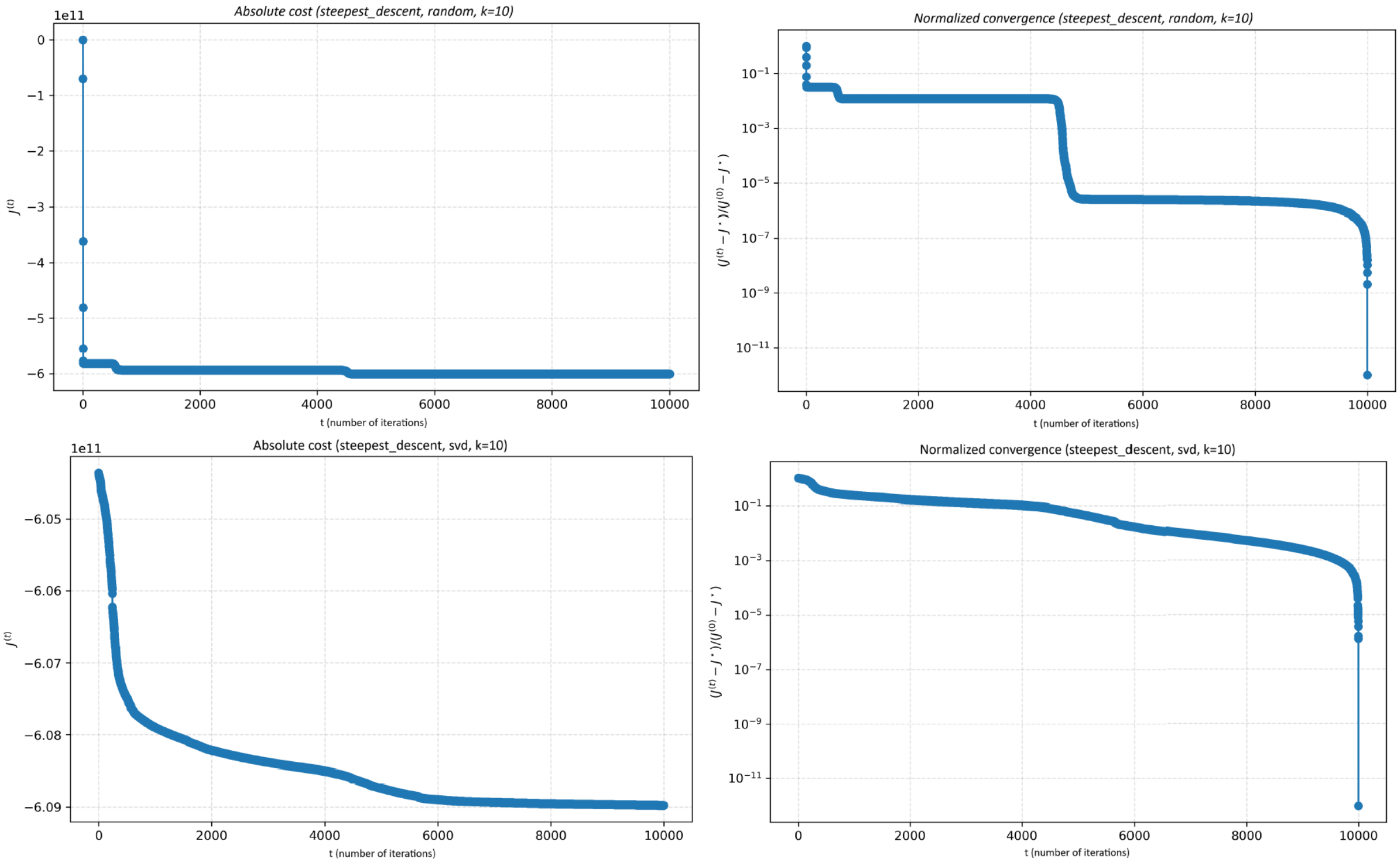

Figure S9: *Convergence behavior of the steepest descent algorithm under different initialization strategies (random vs. SVD).*

Table S1: Overview of gene selection procedures across methods.

Method	Genes selected in benchmark
jsPCA	All genes passing basic prevalence filtering (genes expressed in < 20 cells)
STAGATE	Top 3000 HVGs
SpatialPCA	3000 HVGs, 3000 SVGs, or all genes via an intermediate PCA step (3000 PCs)
GPCA	3000 SVGs selected by SPARK
BASS	3000 SVGs selected by SPARK-X
NichePCA	Removing all genes expressed in < 5 cells
CCST	All input genes (no feature selection step)
Bayespace	Top 2000 HVGs
Spaceflow	Top 3000 HVGs
Leiden	All genes passing basic prevalence filtering (genes expressed in < 20 cells)
Louvain	All genes passing basic prevalence filtering (genes expressed in < 20 cells)

Table S2: Paired t-test comparison of jsPCA against other benchmark methods based on ARI and NMI, using the DLPFC dataset

Metric	Method	p-value	Mean diff
ARI	Bass	0.0748	0.0317
ARI	Bayespace	0.0576	0.0975
ARI	CCST	5.55E-06	0.2317
ARI	GraphPCA	0.0853	0.0550
ARI	Leiden	1.52E-07	0.3508
ARI	Louvain	6.42E-07	0.3408
ARI	NichePCA	3.63E-08	0.3075
ARI	STAGATE	0.0400	0.0542
ARI	Spaceflow	5.83E-07	0.3608
ARI	SpatialPCA	0.6079	-0.0167
NMI	Bass	0.3388	-0.0092
NMI	Bayespace	0.00319	0.1058
NMI	CCST	1.14E-06	0.1967
NMI	GraphPCA	0.6462	0.0083
NMI	Leiden	3.01E-10	0.3750
NMI	Louvain	2.74E-09	0.3692
NMI	NichePCA	2.01E-08	0.1550
NMI	STAGATE	0.3028	0.0183
NMI	Spaceflow	5.70E-08	0.2800
NMI	SpatialPCA	0.8921	-0.0025

Table S3: Results of the Wilcoxon signed-rank test assessing pairwise differences between jsPCA and benchmark methods based on ARI and NMI, using the DLPFC dataset.

Metric	Method	p-value	Median diff
ARI	Bass	0.0791	0.0200
ARI	Bayespace	0.0566	0.1250
ARI	CCST	0.00098	0.2150
ARI	GraphPCA	0.1240	0.0500
ARI	Leiden	0.00049	0.3800
ARI	Louvain	0.00049	0.3750
ARI	NichePCA	0.00049	0.3150
ARI	STAGATE	0.0322	0.0500
ARI	Spaceflow	0.00049	0.3600
ARI	SpatialPCA	0.5088	-0.0200
NMI	Bass	0.1719	0.0200
NMI	Bayespace	0.00098	0.1300
NMI	CCST	0.00049	0.2150
NMI	GraphPCA	0.7109	0.0350
NMI	Leiden	0.00049	0.4200
NMI	Louvain	0.00049	0.4150
NMI	NichePCA	0.00049	0.1800
NMI	STAGATE	0.4502	0.0400
NMI	Spaceflow	0.00049	0.3150
NMI	SpatialPCA	0.5566	0.0050

Table S4: Results of the t-test assessing pairwise differences between jsPCA and benchmark methods based on ARI and NMI, using the first 14 sagittal sections of the MOSTA dataset.

Metric	Method	p-value	Mean diff
ARI	Bass	0.0846	0.0229
ARI	GraphPCA	0.000436	0.0529
ARI	STAGATE	0.000220	0.0500
ARI	SpatialPCA	9.96e-06	0.0936
NMI	Bass	0.0344	0.0214
NMI	GraphPCA	0.000165	0.0336
NMI	STAGATE	0.000122	0.0307
NMI	SpatialPCA	0.000119	0.0479

Table S5: Results of the Wilcoxon signed-rank test assessing pairwise differences between jsPCA and benchmark methods based on ARI and NMI, using the first 14 sagittal sections of the MOSTA dataset.

Metric	Method	p-value	Median diff
ARI	Bass	0.0784	0.025
ARI	GraphPCA	0.00185	0.040
ARI	STAGATE	0.00232	0.050
ARI	SpatialPCA	0.000979	0.085
NMI	Bass	0.0248	0.025
NMI	GraphPCA	0.00160	0.050
NMI	STAGATE	0.00177	0.035
NMI	SpatialPCA	0.00220	0.060

Table S6: Results of the t-test assessing pairwise differences between jsPCA and the benchmark methods (BASS and STAGATE), based on ARI and NMI, using the whole MOSTA dataset.

Metric	Method	p-value	Mean diff
ARI	Bass	1.72e-06	0.0283
ARI	STAGATE	2.91e-14	0.0558
NMI	Bass	4.12e-14	0.0402
NMI	STAGATE	1.63e-14	0.0290

Table S7: Results of the Wilcoxon signed-rank test assessing pairwise differences between jsPCA and the benchmark methods (BASS and STAGATE), based on ARI and NMI, using the whole MOSTA dataset.

Metric	Method	p-value	Median diff
ARI	Bass	1.31e-05	0.020
ARI	STAGATE	1.29e-09	0.050
NMI	Bass	6.25e-09	0.040
NMI	STAGATE	2.59e-09	0.040

Table S8: Ranking of marker genes across jsPCA components, for slice 151673 of the DLPFC dataset.

Gene	jsPCA1	jsPCA2	jsPCA3	jsPCA4	jsPCA5	jsPCA6	jsPCA7	jsPCA8	jsPCA9	jsPCA10
ADCYAP1	2105	257	486	6377	823	447	64	13472	4032	4091
AQP4	236	515	612	8736	11	9629	5499	1631	131	26
BCL11B	7998	13409	833	8959	8110	3826	8458	6793	7908	14586
CUX2	1359	38	232	970	201	14979	12411	7575	9893	1027
HBB	3042	13067	1637	4625	105	153	155	7168	726	27
HPCAL1	1710	2	3026	35	1101	3239	5634	184	1030	3042
IGKC	3372	12218	61	612	43	466	845	1	4	1119
KRT17	2341	327	14	24	27	7929	5	118	9886	8141
MBP	1	671	791	223	1836	4592	12547	10490	1205	6622
MOBP	6	1755	573	754	290	6192	13433	1409	13902	5698
NPY	1358	11356	4071	6842	10682	1279	3379	4450	9401	994
NTNG2	10624	417	93	380	5401	8230	853	4539	12641	8994
PCP4	580	11	7211	13830	527	10	1	147	204	48
RELN	5898	92	13067	1299	80	607	30	709	3643	4684
RORB	1233	88	599	309	9689	11390	12	3831	6925	12476
SNAP25	80	9	580	155	13517	3354	1229	5272	1989	9720
TRABD2A	4689	91	2341	9183	8598	78	7	11778	4915	95

References

Mikhail Belkin and Partha Niyogi. Laplacian eigenmaps for dimensionality reduction and data representation. *Neural computation*, 15(6):1373–1396, 2003.

Junxian Cao, Caifeng Li, Zhao Cui, Shiwen Deng, Tong Lei, Wei Liu, Hongjun Yang, and Peng Chen. Spatial transcriptomics: a powerful tool in disease understanding and drug discovery. *Theranostics*, 14(7):2946, 2024.

Jean-François Cardoso and Antoine Souloumiac. Jacobi angles for simultaneous diagonalization. *SIAM journal on matrix analysis and applications*, 17(1):161–164, 1996.

Ao Chen, Sha Liao, Mengnan Cheng, Kailong Ma, Liang Wu, Yiwei Lai, Xiaojie Qiu, Jin Yang, Jiangshan Xu, Shijie Hao, et al. Spatiotemporal transcriptomic atlas of mouse organogenesis using dna nanoball-patterned arrays. *Cell*, 185(10):1777–1792, 2022.

Carissa Chen, Hani Jieun Kim, and Pengyi Yang. Evaluating spatially variable gene detection methods for spatial transcriptomics data. *Genome Biology*, 25(1):18, 2024.

K Choe, U Pak, Y Pang, W Hao, and X Yang. Advances and challenges in spatial transcriptomics for developmental biology. biomolecules. 2023.

Marco Congedo, Ronald Phlypo, and Dinh-Tuan Pham. Approximate joint singular value decomposition of an asymmetric rectangular matrix set. *IEEE Transactions on Signal Processing*, 59 (1):415–424, 2010.

Kangning Dong and Shihua Zhang. Deciphering spatial domains from spatially resolved transcriptomics with an adaptive graph attention auto-encoder. *Nature communications*, 13(1): 1739, 2022.

Ruben Dries, Qian Zhu, Rui Dong, Chee-Huat Linus Eng, Huipeng Li, Kan Liu, Yuntian Fu, Tianxiao Zhao, Arpan Sarkar, Feng Bao, et al. Giotto: a toolbox for integrative analysis and visualization of spatial expression data. *Genome biology*, 22(1):78, 2021.

Ian K Gingerich, Brittany A Goods, and H Robert Frost. Randomized spatial pca (rasp): a computationally efficient method for dimensionality reduction of high-resolution spatial transcriptomics data. *Research Square*, pages rs–3, 2025.

Clara Guijarro, Solène Song, Benoit Aigouy, Raphaël Clément, Paul Villoutreix, and Robert G Kelly. Single-cell morphometrics reveals t-box gene-dependent patterns of epithelial tension in the second heart field. *Nature Communications*, 15(1):9512, 2024.

Laleh Haghverdi, Aaron TL Lun, Michael D Morgan, and John C Marioni. Batch effects in single-cell rna-sequencing data are corrected by matching mutual nearest neighbors. *Nature biotechnology*, 36(5):421–427, 2018.

Minsheng Hao, Kui Hua, and Xuegong Zhang. Somde: a scalable method for identifying spatially variable genes with self-organizing map. *Bioinformatics*, 37(23):4392–4398, 2021a.

Yuhan Hao, Stephanie Hao, Erica Andersen-Nissen, William M Mauck, Shiwei Zheng, Andrew Butler, Maddie J Lee, Aaron J Wilk, Charlotte Darby, Michael Zager, et al. Integrated analysis of multimodal single-cell data. *Cell*, 184(13):3573–3587, 2021b.

Trevor Hastie, Robert Tibshirani, Jerome Friedman, et al. The elements of statistical learning, 2009.

Jian Hu, Xiangjie Li, Kyle Coleman, Amelia Schroeder, Nan Ma, David J Irwin, Edward B Lee, Russell T Shinohara, and Mingyao Li. Spagcn: Integrating gene expression, spatial location and histology to identify spatial domains and spatially variable genes by graph convolutional network. *Nature methods*, 18(11):1342–1351, 2021.

Yang Jin, Yuanli Zuo, Gang Li, Wenrong Liu, Yitong Pan, Ting Fan, Xin Fu, Xiaojun Yao, and Yong Peng. Advances in spatial transcriptomics and its applications in cancer research. *Molecular Cancer*, 23(1):129, 2024.

T Jombart, S Devillard, Anne-Béatrice Dufour, and D Pontier. Revealing cryptic spatial patterns in genetic variability by a new multivariate method. *Heredity*, 101(1):92–103, 2008.

Jan Lause, Philipp Berens, and Dmitry Kobak. Analytic pearson residuals for normalization of single-cell rna-seq umi data. *Genome biology*, 22(1):258, 2021.

Richard B Lehoucq, Danny C Sorensen, and Chao Yang. *ARPACK users' guide: solution of large-scale eigenvalue problems with implicitly restarted Arnoldi methods*. SIAM, 1998.

Jiachen Li, Siheng Chen, Xiaoyong Pan, Ye Yuan, and Hong-bin Shen. Ccst: Cell clustering for spatial transcriptomics data with graph neural network. 2021.

Zheng Li and Xiang Zhou. Bass: multi-scale and multi-sample analysis enables accurate cell type clustering and spatial domain detection in spatial transcriptomic studies. *Genome biology*, 23 (1):168, 2022.

Wendi Liu, Anusha Puri, Doris Fu, Lee Chen, Cassia Wang, Manolis Kellis, and Jiekun Yang. Dissecting the tumor microenvironment in response to immune checkpoint inhibitors via single-cell and spatial transcriptomics. *Clinical & Experimental Metastasis*, 41(4):313–332, 2024.

Zhaoyang Liu, Dongqing Sun, and Chenfei Wang. Evaluation of cell-cell interaction methods by integrating single-cell rna sequencing data with spatial information. *Genome Biology*, 23(1): 218, 2022.

Ikmal Maulana and Rusdianto Roestam. Optimizing knn algorithm using elbow method for predicting voter participation using fixed voter list data (dpt). *Jurnal Sosial Teknologi*, 4(7): 441–451, 2024.

Kristen R Maynard, Leonardo Collado-Torres, Lukas M Weber, Cedric Uytingco, Brianna K Barry, Stephen R Williams, Joseph L Catallini, Matthew N Tran, Zachary Besich, Madhavi Tippani, et al. Transcriptome-scale spatial gene expression in the human dorsolateral prefrontal cortex. *Nature neuroscience*, 24(3):425–436, 2021.

Valeria Montano and Thibaut Jombart. An eigenvalue test for spatial principal component analysis. *BMC bioinformatics*, 18:1–7, 2017.

Lambda Moses and Lior Pachter. Museum of spatial transcriptomics. *Nature methods*, 19(5): 534–546, 2022.

Michelli Faria de Oliveira, Juan Pablo Romero, Meii Chung, Stephen R Williams, Andrew D Gottscho, Anushka Gupta, Susan E Pilipauskas, Seayar Mohabbat, Nandhini Raman, David J Sukovich, et al. High-definition spatial transcriptomic profiling of immune cell populations in colorectal cancer. *Nature Genetics*, pages 1–12, 2025.

Jia-Min B Pang, David J Byrne, Alice RT Bergin, Franco Caramia, Sherene Loi, Kylie L Gorringe, and Stephen B Fox. Spatial transcriptomics and the anatomical pathologist: Molecular meets morphology. *Histopathology*, 84(4):577–586, 2024.

Krzysztof Polański, Raquel Bartolomé-Casado, Ioannis Sarropoulos, Chuan Xu, Nick England, Frode L Jahnsen, Sarah A Teichmann, and Nadav Yayon. Bin2cell reconstructs cells from high resolution visium hd data. *Bioinformatics*, 40(9):btae546, 2024.

Anjali Rao, Dalia Barkley, Gustavo S França, and Itai Yanai. Exploring tissue architecture using spatial transcriptomics. *Nature*, 596(7871):211–220, 2021.

H Ren, BL Walker, Z Cang, and Q Nie. Identifying multicellular spatiotemporal organization of cells with spaceflow. nat. commun. 13, 4076, 2022.

Douglas A Reynolds et al. Gaussian mixture models. *Encyclopedia of biometrics*, 741(659-663):3, 2009.

Zhihan Ruan, Weijun Zhou, Hong Liu, Jinmao Wei, Yichen Pan, Chaoyang Yan, Xiaoyi Wei, Wenting Xiang, Chengwei Yan, Shengquan Chen, et al. Precise detection of cell-type-specific domains in spatial transcriptomics. *Cell Reports Methods*, 4(8), 2024.

Abhishek Sampath Kumar, Luyi Tian, Adriano Bolondi, Amèlia Aragonés Hernández, Robert Stickels, Helene Kretzmer, Evan Murray, Lars Wittler, Maria Walther, Gabriel Barakat, et al. Spatiotemporal transcriptomic maps of whole mouse embryos at the onset of organogenesis. *Nature Genetics*, 55(7):1176–1185, 2023.

Dominik Saul and Robyn Laura Kosinsky. Spatial transcriptomics herald a new era of transcriptome research. *Clinical and Translational Medicine*, 13(5):e1264, 2023.

Darius P Schaub, Behnam Yousefi, Nico Kaiser, Robin Khatri, Victor G Puelles, Christian F Krebs, Ulf Panzer, and Stefan Bonn. Pca-based spatial domain identification with state-of-the-art performance. *Bioinformatics*, 41(1):btaf005, 2025.

Lulu Shang and Xiang Zhou. Spatially aware dimension reduction for spatial transcriptomics. *Nature communications*, 13(1):7203, 2022.

Shiquan Sun, Jiaqiang Zhu, and Xiang Zhou. Statistical analysis of spatial expression patterns for spatially resolved transcriptomic studies. *Nature methods*, 17(2):193–200, 2020.

Valentine Svensson, Sarah A Teichmann, and Oliver Stegle. Spatialde: identification of spatially variable genes. *Nature methods*, 15(5):343–346, 2018.

Fabian J Theis, Thomas P Cason, and P-A Absil. Soft dimension reduction for ica by joint diagonalization on the stiefel manifold. In *International Conference on Independent Component Analysis and Signal Separation*, pages 354–361. Springer, 2009.

James Townsend, Niklas Koep, and Sebastian Weichwald. Pymanopt: A python toolbox for optimization on manifolds using automatic differentiation. *Journal of Machine Learning Research*, 17(137):1–5, 2016.

Vincent A Traag, Ludo Waltman, and Nees Jan Van Eck. From louvain to leiden: guaranteeing well-connected communities. *Scientific reports*, 9(1):1–12, 2019.

Katy Vandereyken, Alejandro Sifrim, Bernard Thienpont, and Thierry Voet. Methods and applications for single-cell and spatial multi-omics. *Nature Reviews Genetics*, 24(8):494–515, 2023.

Lukas M Weber, Arkajyoti Saha, Abhirup Datta, Kasper D Hansen, and Stephanie C Hicks. nnsvg for the scalable identification of spatially variable genes using nearest-neighbor gaussian processes. *Nature communications*, 14(1):4059, 2023.

F Alexander Wolf, Philipp Angerer, and Fabian J Theis. Scanpy: large-scale single-cell gene expression data analysis. *Genome biology*, 19:1–5, 2018.

Chenglong Xia, Jean Fan, George Emanuel, Junjie Hao, and Xiaowei Zhuang. Spatial transcriptome profiling by merfish reveals subcellular rna compartmentalization and cell cycle-dependent gene expression. *Proceedings of the National Academy of Sciences*, 116(39):19490–19499, 2019.

Zhicheng Xu, Weiwen Wang, Tao Yang, Ling Li, Xizheng Ma, Jing Chen, Jieyu Wang, Yan Huang, Joshua Gould, Huifang Lu, et al. Stomicsdb: a comprehensive database for spatial transcriptomics data sharing, analysis and visualization. *Nucleic acids research*, 52(D1): D1053–D1061, 2024.

Guanao Yan, Shuo Harper Hua, and Jingyi Jessica Li. Categorization of 34 computational methods to detect spatially variable genes from spatially resolved transcriptomics data. *Nature Communications*, 16(1):1141, 2025.

Jiyuan Yang, Lu Wang, Lin Liu, and Xiaoqi Zheng. Graphpca: a fast and interpretable dimension reduction algorithm for spatial transcriptomics data. *Genome Biology*, 25(1):287, 2024.

Zhiyuan Yuan, Fangyuan Zhao, Senlin Lin, Yu Zhao, Jianhua Yao, Yan Cui, Xiao-Yong Zhang, and Yi Zhao. Benchmarking spatial clustering methods with spatially resolved transcriptomics data. *Nature Methods*, 21(4):712–722, 2024.

Edward Zhao, Matthew R Stone, Xing Ren, Jamie Guenthoer, Kimberly S Smythe, Thomas Pulliam, Stephen R Williams, Cedric R Uytingco, Sarah EB Taylor, Paul Nghiem, et al. Spatial transcriptomics at subspot resolution with bayesspace. *Nature biotechnology*, 39(11):1375–1384, 2021.

Ran Zhou, Gaoxia Yang, Yan Zhang, and Yuan Wang. Spatial transcriptomics in development and disease. *Molecular Biomedicine*, 4(1):32, 2023.

Jiaqiang Zhu, Shiquan Sun, and Xiang Zhou. Spark-x: non-parametric modeling enables scalable and robust detection of spatial expression patterns for large spatial transcriptomic studies. *Genome biology*, 22(1):184, 2021.
